# A Cyclic Permutation Approach to Removing Spatial Dependency between Clustered Gene Ontology Terms

**DOI:** 10.3390/biology13030175

**Published:** 2024-03-08

**Authors:** Rachel Rapoport, Avraham Greenberg, Zohar Yakhini, Itamar Simon

**Affiliations:** 1 Microbiology and Molecular Genetics, Hebrew University of Jerusalem-IMRIC, Jerusalem 9112102, Israel; 2 Efi Arazi School of Computer Science, Reichman University (IDC Herzliya), Herzliya 4610101, Israel; 3 Department of Computer Science, Technion-Israel Institute of Technology, Haifa 3200003, Israel

**Keywords:** gene set enrichment analysis (GSEA), GO annotations, spatial dependencies, cyclic permutation, replication timing, copy number alterations (CNA)

## Abstract

**Simple Summary:**

In the intricate field of genomic research, researchers frequently look for the enrichment of genes with a common function. Traditionally, genes are analyzed as if they function independently. However, this assumption may not hold true in large genomic regions, where genes with similar functions exist in close proximity and may influence each other. Our research introduces an advanced method to discern whether the observed patterns in gene groups are due to their spatial closeness, or stem from other biological factors. This approach is particularly crucial in studying large genomic loci, where conventional methods might overlook the nuanced interplay of functionally similar genes. By implementing our technique, we significantly enhance the precision of genomic analyses, particularly in these extensive areas. This advancement is vital as it deepens our understanding of gene interactions within large genomic regions.

**Abstract:**

Traditional gene set enrichment analysis falters when applied to large genomic domains, where neighboring genes often share functions. This spatial dependency creates misleading enrichments, mistaking mere physical proximity for genuine biological connections. Here we present Spatial Adjusted Gene Ontology (SAGO), a novel cyclic permutation-based approach, to tackle this challenge. SAGO separates enrichments due to spatial proximity from genuine biological links by incorporating the genes’ spatial arrangement into the analysis. We applied SAGO to various datasets in which the identified genomic intervals are large, including replication timing domains, large H3K9me3 and H3K27me3 domains, HiC compartments and lamina-associated domains (LADs). Intriguingly, applying SAGO to prostate cancer samples with large copy number alteration (CNA) domains eliminated most of the enriched GO terms, thus helping to accurately identify biologically relevant gene sets linked to oncogenic processes, free from spatial bias.

## 1. Introduction

An essential practice in the analysis of high-throughput biological data involves identifying enriched genes within pre-defined gene sets, such as those defined by the Gene Ontology (GO) project [1]. Various tools have been developed to perform such an enrichment analysis [2,3]. Enrichment analysis allows for the inference of the functions of co-expressed genes. For instance, when GO terms are enriched in a set of over-expressed genes, it suggests potential functional pathways activated under those conditions. The statistical significance of enrichment is usually determined using the hypergeometric test (Fisher exact test), Chi-Square, or binomial distribution tests [4,5]. The underlying assumption in all these tests is the independence between the identified genes. This means that each gene has an equal probability of being a member of a selected list of genes, and choosing one gene from a gene set does not affect the likelihood of choosing another gene from the same set. This is a crucial assumption for assessing whether the observed enrichment could have occurred spuriously.

While such an assumption is reasonable for expression profiling results, where each gene is measured separately, it becomes less obvious in cases involving larger genomic regions that contain many genes. For example, replication domains are large (median size of 0.4–0.8 Mb [6]) yet it is common practice to determine the function of genes within early or late replicating domains using gene set enrichment analyses [7,8]. In such cases, all the genes within the replication domain are included in the analysis, even though they may no longer be independent. Were the distribution of genes in the genome random, the proximity between genes would not violate the gene independence assumption. However, this is not the case, as there are clear functional dependencies between adjacent genes [9,10,11,12]. In many species, it has been shown that co-expressed genes tend to cluster in the genome [13,14]. For instance, in the human genome, housekeeping genes have been found in clusters [15]. Additionally, functionally related genes also tend to be clustered. An analysis of KEGG pathways in five eukaryotes revealed a high proportion of gene clustering for those sharing the same pathways [16]. Similarly, an analysis of the clustering of GO terms revealed that clusters of functionally related genes are common, not only in bacterial operons but also in *H. sapiens*, *Mus musculus*, *S. cerevisiae*, *C. elegans*, *D. melanogaster*, and *Arabidopsis thaliana* [17].

A similar problem exists in the interpretation of genome-wide association studies’ (GWASs’) results due to the linkage disequilibrium structure of SNPs, and to the clustering of functionally related elements in the genome. Cabrera et al. [18] developed a method using cyclic permutations to address dependencies between SNPs and adjacent genes. However, their method was developed specifically for GWASs, and does not address a more general issue of gene enrichment analysis.

Here, we expand the cyclic permutation approach to address the spatial dependency problem in the context of gene set enrichment analyses. We compared the list of enriched genes to random gene lists generated through cyclic permutations, thereby preserving the spatial dependencies between genes. To accommodate this change, we replaced the commonly used statistics with a sampling method that covers all possible cyclic permutations. We applied our novel approach to various examples of genomic experiments that were designed to identify large genomic domains including replication timing (RT), lamin-associated domains, large H3K27me3 and H3K9me3 domains, HiC compartments and copy number regional alterations in cancer. Our approach allows for the cleaning of the list of enriched GO terms, removing terms that were enriched solely due to the genomic co-location of the genes in the term. Overall, our approach overcomes the dependency problem and distinguishes between enrichments that are due only to GO term clustering and those that are more likely due to the biology of the analyzed domains.

## 2. Methods

All analyses and the SAGO pipeline were conducted using R (version 4.3.2), a language and environment for statistical computing, utilizing the ggplot2 package (version 3.3.6) for figure generation.

### 2.1. SAGO Pipeline

The SAGO pipeline employs a distinctive cyclic permutation strategy to statistically assess gene associations within specified genomic intervals at the gene level. This process begins by identifying the transcription start sites (TSS) of genes within input intervals using the GenomicFeatures package (version 1.44.2) and the Bioconductor TxDb object for the organism and genomic build. Counts for the corresponding Gene Ontology (GO) terms for each gene were determined using the AnnotationDbi package (version 1.54.1) and the Bioconductor orgDb database. For each GO Term, all its ancestral parents were identified and included in the analysis using the GOfuncR package (version 1.12.0).

### 2.2. Hypergeometric Test for GO Term Enrichment

To estimate the enrichment of GO terms, the hypergeometric test was utilized to assess whether the observed frequency of specific GO terms in our gene subset significantly exceeds chance expectations. This test was performed using the phyper function in R, calculating a hypergeometric *p*-value for each GO term to gauge enrichment significance. The Benjamini-Hochberg correction was applied to adjust for multiple hypothesis testing.

### 2.3. Cyclic and Random Permutations

Significant GO terms (FDR adjusted *p*-value < 0.1) from the hypergeometric test were further analyzed using both cyclic and random permutations. The cyclic permutation approach maintains the genome’s spatial integrity by treating it as circular and systematically ordering genes by chromosome and location. We iteratively performed n-1 cyclic permutations, where n is the total number of genes in the genome. In each permutation, gene positions were incrementally shifted while preserving their order.

Concurrently, random permutations were also executed, where the intervals were randomly populated with genes. In these random permutations, the spatial ordering of the genes is not preserved, providing a contrast to the cyclic approach.

For both permutation methods, gene counts associated with significant GO terms were recounted, and experimental *p*-values were calculated based on the frequency of permutations showing an at-least-as-equal enrichment of a GO term as in the actual data. These *p*-values were then corrected for multiple hypotheses using the Bonferroni correction.

### 2.4. Linear Regression Analysis

We performed a linear regression between the results of the cyclic and random permutations for each GO term. The model and its residuals were calculated using the lm function.

### 2.5. Random Intervals Analysis

**Figure 1:**
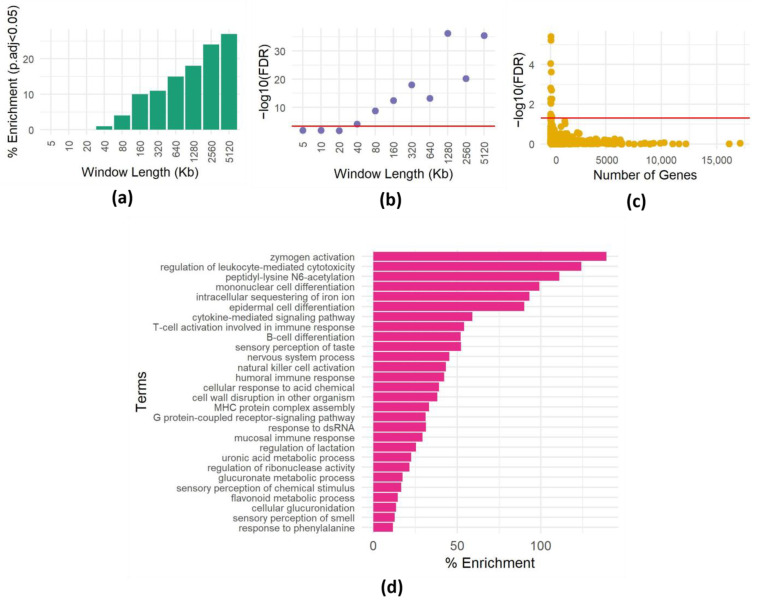
Impact of spatial dependencies on enrichment analysis. (**a**) Enrichment of the GO term “detection of stimulus involved in sensory perception of smell” (GO:0007608) in 400 randomly chosen genomic intervals of varying lengths. The *y*-axis shows the percentage of times the GO term was found to be enriched at a significance level of *p* < 0.0005 (equivalent to *p* < 0.05 with Bonferroni correction for 100 hypotheses) out of 100 repetitions. The *x*-axis shows the length of the genomic intervals in kilobases (Kb). (**b**) Minimum *p*-value for enrichment of the GO term “detection of stimulus involved in sensory perception of smell” (GO:0007608) in 400 randomly chosen genomic intervals of varying lengths. The *y*-axis shows the minimum adjusted *p*-value observed out of 100 repetitions. The *x*-axis shows the length of the genomic intervals in kilobases (Kb). The red line is at a significance level of adjusted *p* < 0.0005. (**c**) Enrichment results for one set of 400 randomly chosen genomic intervals of 500 Kb each. For each term, the -log FDR corrected *p*-value is plotted as a function of the size (number of genes) of the term. The red line is drawn at FDR = 0.05. (**d**) Heatmap showing the frequency of enrichment of GO terms in 400 randomly chosen genomic intervals of 500 Kb each. The *y*-axis shows the GO terms. The *x*-axis shows the percentages of enrichment of 100 repetitions (the full list is shown in Table S1).

**Figure 2:**
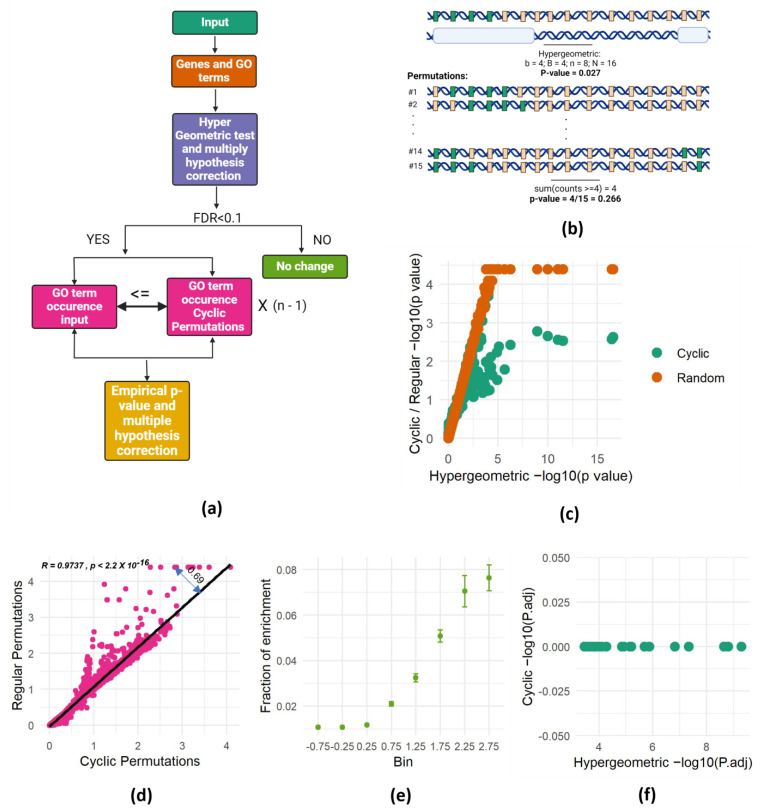
SAGO corrects successfully spatial dependencies. (**a**) Schematic representation of SAGO. (**b**) Schematic representation of the cyclic permutations approach. This figure depicts the relationship between genes and their association with a specific Gene Ontology (GO) term, using two complementary panels. The top panel presents a genomic landscape, where green rectangles represent genes linked to the GO term of interest, and gray rectangles represent the remaining genes. Below this landscape, white bars highlight the specific genomic intervals measured in an experiment. A hypergeometric *p*-value, calculated based on the observed enrichment of GO-associated genes within the measured intervals, is shown beneath the panels. The bottom panel showcases cyclic permutations of the genes involved in the experiment. Each subsequent row represents a different permutation, with the genes re-ordered in a circular fashion. The *p*-value shown below the last permutation row highlights the overall probability of observing at least four GO-associated genes in any of the n-1 possible cyclic permutations. (**a**,**b**) were created with BioRender.com (accessed on 3 March 2024). (**c**) Scatter plot of one of the random intervals set, showing the *p*-value of each term calculated either by the hypergeometric test (X-axis) or by performing 21,633 permutations (cyclic—green; random—brown). (**d**) Scatter plot showing the association between *p*-values (−log) obtained by cyclic and regular permutations. A linear regression line and the distance of one dot from it (residual value) are shown. (**e**) For each term, the fraction of runs that it was enriched (out of 100 random runs) is shown. All the terms were separated into eight bins according to their residual value ± 0.25 (X-axis) and the mean and standard error of each bin are shown. (**f**) Hypergeometric- versus cyclic-adjusted *p*-values are shown for all terms enriched in one set of random intervals (shown in Figure 1c).

Random regions for Figure 1 and Figure 2 were sampled using the regioneR package (version 1.22.0, Ref. [19]). In Figure 2e, for each term that was enriched at least in one random dataset, we calculated the fraction of random runs in which it was enriched (Bonferroni *p*-adjust value < 0.05). Each term was assigned to a bin according to the average residual value over the 100 random runs. The average number and the standard error were calculated for each bin. For Figure S1, the bins were calculated in the same way as in Figure 2e, and the fraction of correction is the fraction of the enriched GO terms in each bin which, after applying SAGO, had a Bonferroni adjusted *p*-value > 0.05.

### 2.6. Data Sources and Processing

As stated in the Results section, we were interested in exploring the utility of SAGO on experiments that result in large genomic intervals. For each type of experiment, we chose a reliable dataset from the literature.

H3K27me3 and H3K9me3 datasets were downloaded from the ENCODE portal (https://www.encodeproject.org/, accessed on 16 November 2023) using the following identifiers: ENCFF803QFK, ENCFF277EYC. Broad genomic domains were identified using the RECOGNICER pipeline [20].

Liver LAD data are available under accession GSM5669232.

Hi-C data for ESC and NPC can be accessed in the GEO database under accession code GSE96107. Differential B compartments were identified using the dcHiC pipeline [21].

Replication timing for primordial germ cells and mouse embryonic fibroblasts can be found under the accession GSE109804. Determination of differential regions was performed following the methods described in [22].

All other datasets used in this paper are provided as Supplementary Materials in the corresponding manuscripts.

## 3. Results

### 3.1. Spatial Dependencies Affect Enrichment Analyses

By definition, a list of randomly selected genes should not show an enrichment of any GO category. Similarly, analyzing randomly chosen genomic intervals for gene enrichment should not reveal any GO terms, unless genes within the term share spatial dependencies.

To investigate the presence of spatial dependencies and the need to correct for them, we focused on the “sensory perception of smell” GO term (GO:0007608), containing 894 genes mostly clustered in a few genomic loci [23]. We conducted a series of experiments to assess how often we could observe an enrichment of genes belonging to this category by chance. We sampled 400 random genomic intervals of various lengths and assessed the enrichment of the GO term using the hypergeometric test (see Methods). We repeated this 100 times, recording the instances of significant enrichment (*p* < 0.0005, equivalent to *p* < 0.05 after Bonferroni correction for 100 hypotheses) for each interval size (Figure 1a). Surprisingly, even in relatively small windows (40 Kb), the GO term showed enrichment multiple times (Figure 1a), and the minimum *p*-value (among the 100 repeats) reached very low values (Figure 1b).

To further explore this phenomenon, we extended the analysis to include all GO terms within 400 windows, each 500 Kb. Many GO terms surpassed the enrichment threshold (Figure 1c; significance threshold Bonferroni corrected *p*-value < 0.05). Interestingly, enriched GO terms have various sizes (numbers of genes), implying that genomic dispersion, not term size, contributes to these spurious enrichments. This observation underscores the critical role of spatial gene dependencies within regions in generating misleading enrichments, emphasizing the need to address spatial correlations in enrichment analyses. Furthermore, repeating the procedure of choosing random regions 100 times allows us to identify GO terms that are repeatedly enriched in random regions (Figure 1d), suggesting that these terms are not randomly distributed in the genome.

### 3.2. Developing the Spatial Adjusted Gene Ontology (SAGO) Analysis Tool

The conventional approach for calculating enrichment for a given list of genomic loci involves finding the genes within each interval, assigning their corresponding GO terms, and statistically comparing the number of genes in each GO term to a background set, typically containing all the measured genes. This method, however, disregards the spatial dependency between genes, assuming the probability of a gene being associated with a particular GO term solely depends on the total number of genes in the genome belonging to that specific GO term.

To address the spatial dependencies problem in GO enrichment analyses, we adopted the cyclic permutation approach [18], which preserved the spatial dependency between genes in the background set. Instead of comparing the number of identified genes within each GO term to its frequency across the entire genome, we compared it to the number of genes in each term in all possible permutations that preserved the genomic spatial dependency. This means that we counted the number of genes of each GO term falling within the genomic intervals of interest and compared it to the number of genes from the same term falling within these intervals in all possible permutations. The permutations were done using a cyclic permutation scheme, where the order of genes in the genome is maintained but a different set of genes populates the intervals in each permutation. These permuted genomes serve as the background against which we assess enrichment. The experimental *p*-values are computed by calculating the fraction of permutations in which the number of genes from a certain GO category falling within the intervals was at least equal to the number observed in the actual experiment (Figure 2a,b).

While the cyclic permutation approach preserves gene order, it has a finite number of distinct permutations. For a genome with *n* genes, only *n*-1 unique permutations exist. Therefore, for each experiment, we conducted all possible distinct permutations and calculated the fraction of permutations that resulted in at least as many genes as the observed number of genes from each GO term intersecting the interval of interest. This proportion becomes the bootstrapped *p*-value, representing the probability of randomly obtaining at least the observed number of genes from a specific GO category within the actual gene order of the analyzed genome (Figure 2b). This approach effectively addresses spatial dependencies and offers a robust method for assessing spatial enrichment in gene set analysis.

To validate the effectiveness of SAGO, we revisited the experiment presented in Figure 1c, this time calculating the *p*-values using both cyclic and random permutations. This analysis revealed that the *p*-values obtained through the permutation-based methodology closely resemble those of the hypergeometric test, with low *p*-values plateauing due to the finite number of random permutations. This finding confirms the accuracy of our *p*-value calculations in SAGO, supporting its validity as a robust method for assessing spatial enrichment compared to conventional approaches. (Figure 2c).

Comparing *p*-values from cyclic and random permutations revealed striking differences. While most terms yielded similar values with both methods, some exhibited significantly lower *p*-values in random permutations. We suspected that the degree of deviation from the regression line captured the spatial dependency between the genes in each GO term. Thus, we calculated the distance from the regression line (double-headed arrow in Figure 2d) and compared it to the chance for each term to be enriched in random intervals. To this end, we used the 100 repeats of the random intervals to generate a chance score for each term. As expected, terms with high average residual values appeared to be enriched in many random permutations (Figure 2e), suggesting the residual value as a reliable indicator for spatial dependency within a GO term.

### 3.3. Multiple Hypothesis Corrections

Our experimental *p*-values are constrained by the number of possible permutations. For a typical mammalian genome with ~20,000 genes, the lowest achievable *p*-value is 1/20,000 = 5 × 10^−5^. Such *p*-values are usually not sufficiently small enough to sustain a multiple hypothesis correction of thousands of hypotheses (the typical range of GO terms or other sets tested in each experiment). To overcome this limitation and ensure a robust enrichment assessment, SAGO employs a two-step sequential multiple hypothesis correction [24]. First, we apply an FDR correction on the hypergeometric *p*-values from the standard enrichment analysis. Only terms exceeding this initial FDR threshold of 0.1 are then subjected to the more stringent cyclic permutation test. We note that, for random data, we will have no results exceeding the FDR = 0.1 threshold. Hypotheses reaching this threshold due to genomic proximity will be filtered out as potential false positives in the second step. This approach reduces the number of hypotheses by up to two orders of magnitudes, further reinforced by correcting for multiple hypotheses using the Bonferroni approach.

Applying SAGO on the random dataset described in Figure 1c successfully eliminated all enrichments (Figure 2f), strongly suggesting that the enrichments observed in the random dataset were attributable to the spatial proximity among genes sharing the same GO term. Furthermore, applying SAGO on all 100 random datasets revealed that SAGO effectively eliminated all GO terms with residual values exceeding one (Figure S1).

### 3.4. Applying SAGO to Replication Timing Data

Next, we applied SAGO to actual experimental data, particularly focusing on replication timing (RT) data. Replication timing domains are characterized by large intervals (with a median size of 400–800 Kb; Ref. [6]). Given the large size of RT domains, enrichment analysis is prone to the biases SAGO aims to address.

**Figure 3:**
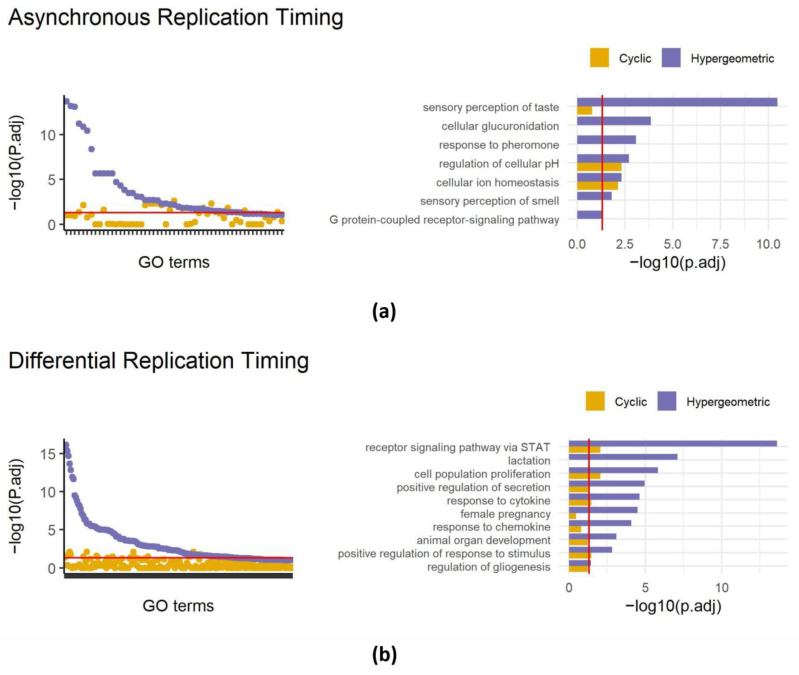
Applying SAGO to replication timing data. Left panels—dot plots showing adjust *p*-values of terms enriched in regions showing asynchronous replication (**a**) or differential replication timing between MEFs and primordial germ cells (**b**). For each term the cyclic permutation (yellow) and the naïve hypergeometric (blue) adjusted *p*-values are shown. The red line is drawn at an adjusted *p*-value = 0.05. Right panels—bar graphs comparing cyclic permutation and hypergeometric adjusted *p*-values for selected GO terms (the full list is shown in Table S2), for asynchronous replication regions (**a**) and differential regions (**b**). The red line is drawn at an adjusted *p*-value = 0.05.

In our recent work, we identified approximately 400 genomic intervals that replicate asynchronously in the mouse genome, covering 226 Mb. A regular gene set enrichment analysis of these intervals revealed 42 enriched terms including the sensory perception of taste and the sensory perception of smell (also present in random datasets, Figure 1d). Reanalyzing with SAGO eliminated 26 out of the 42 enriched terms (Figure 3a), including all terms associated with the cellular perception of taste and smell and the response to pheromones. Intriguingly, terms associated with ion homeostasis and the regulation of cellular pH, remained significant, suggesting that the latter categories are enriched independently from their genomic distribution (Figure 3a and Table S2).

Similarly, analyzing regions with differential replication timing between primordial germ cells and mouse embryonic fibroblasts [22] revealed the enrichment of 139 GO terms, surprisingly including lactation, female pregnancy, and the response to chemokine. Applying SAGO eliminated 122 GO terms, including the aforementioned terms, while preserving the response to cytokine, cell fate determination, epithelial cell proliferation and others. (Figure 3b and Table S2).

### 3.5. Expanding the Use of SAGO to Additional Types of Data

**Figure 4:**
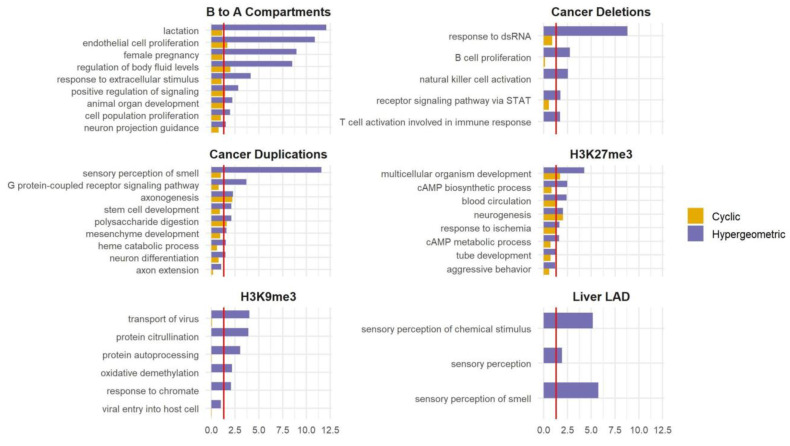
Applying SAGO to other datasets. Bar graphs comparing the cyclic permutation (yellow) and the hypergeometric (blu) corrected *p*-value for selected GO terms (the full list is shown in Table S2), for regions that changed from compartment B to A upon ES differentiation to NPC; regions deleted or duplicated in prostate cancer; large H3K27me3 and H3K9me3 ChIP-Seq domains in the liver tissue of a male adult and heart tissue embryo cells, respectively; and liver LAD. The red line is drawn at an adjusted *p*-value = 0.05.

SAGO’s utility extends beyond RT data. Any regional measurements ideally require a spatial adjustment of the type that SAGO provides. We applied SAGO on selected datasets that capture large genomic regions including large (10^6^–10^7^ bp) H3K27me3 and H3K9me3 domains obtained by ChIP-seq [25,26,27], regions transitioning from compartment B to A upon ES differentiation to NPC as determined by HiC data [28], lamin-associated domains (LADs) in the liver [29] and regions with copy number alterations observed in cancer patients [30]. In all cases, SAGO eliminated most enriched terms, especially those lacking intuitive justifications. This helped highlight terms whose enrichment is not a consequence of the genomic spatial distribution of the genes within the term (Figure 4 and Table S2). For example, in the B to A compartments, terms associated with lactation were eliminated, while terms associated with endothelial and epithelial cell proliferation remained. In the liver LADs, the “sensory perception of smell” category was eliminated. In the regions deleted in patients SP102620 and SP102622, all enriched terms were eliminated. In the regions duplicated in patient SP102622, many categories associated with sensory perception and neuronal development were eliminated, yet categories associated with synapse and axon guidance remained. In the ChIP-seq data, all H3K9me3-enriched terms were eliminated, whereas many of the H3K27me3-enriched terms were retained.

## 4. Discussion

In genomic data analysis, a common approach involves identifying gene set enrichments within a list of genes obtained as a result of a measurement or an experiment. For instance, in RNA-seq experiments, researchers often find a list of differentially expressed genes and then employ gene set enrichment analysis to determine if this list is enriched with specific types of genes [3,31,32]. This analysis implicitly assumes gene independence, meaning finding one gene does not influence others, unless a shared biological process is at play under the studied conditions.

However, the gene-independence assumption can be shaky, especially when measuring large genomic domains. In such cases, assuming all genes within the identified domain are independent can be misleading. To address this, we developed a permutation-based method. This method compares the observed enrichment to the enrichment obtained in control sets that maintain the genomic composition and order, effectively accounting for spatial dependencies between genes.

Our cyclic permutations approach compares a set of enriched genes within genomic domains to all possible shifted versions of the set, still fitting within the domains and preserving the original genomic organization. This effectively eliminates enrichments that might arise in random sets of intervals due to the clustering of certain Gene Ontology (GO) terms in the genome, by taking into consideration the genome structure (see Figure 1 and Figure 2f for comparison). Thus, in every case in which the spatial dependency between adjacent genes may be an issue, SAGO should be considered.

Our cyclic permutations-based approach has two main drawbacks worth noting. First, it is resource intensive as it involves calculating GO term enrichments for approximately 20,000 permutations. Secondly, its statistical power is limited due to the restricted number of cyclic permutations (*n* − 1, where *n* is the number of genes in the genome), resulting in a minimal *p*-value of 1/*n* − 1, which is equal to 5 × 10^−5^ for a typical mammalian genome with around 20,000 genes. These limited *p*-values can pose challenges, particularly when performing multiple hypothesis corrections.

To address the limited *p*-value issue, we applied SAGO only on terms that passed an initial hypergeometric test (FDR < 0.1), thereby reducing the number of hypotheses tested. This two-stepped test, where only terms that passed an initial test (corrected for multiple hypotheses) are used in the second test, has been previously applied in the context of GWAS and SNP analyses [24].

To quantify the spatial dependency of each term, we calculated a metric based on the deviation of its *p*-values from the linear regression line between random and cyclic permutations (Figure 2d). Indeed, terms with high residual values exhibit more frequent enrichment in random data (Figure 2e). Terms with low residual values (<0.5) typically retain significance after applying SAGO, while those with high residual values (>1) are almost always eliminated (Figure S1).

Applying SAGO reduces many of the terms that were found significant due to the spatial dependency between genes. However, it also reduces the *p*-values due to the limited statistical power of our permutation-based technique. Consequently, the balance between false positive and false negative results is shifted. Using the residual metric can be useful in determining the set of terms that were eliminated due to the spatial dependencies and not due to the statistical power.

SAGO’s strength has been demonstrated in the context of replication timing data (Figure 3), where large genomic regions with similar RT values are present, and in other types of genomic datasets that involve large genomic domains (Figure 4). It is particularly relevant when the measured intervals exceed the distances between genes, increasing the likelihood of multiple genes being contained within the intervals. In such situations, which are common in studying large genomic domains, but also relevant to CRISPR screens results [33], SAGO is essential because the assumption of conventional statistics (that there is independence between genes) does not hold. We have demonstrated this with replication timing, compartments, copy number alterations, LADs and large closed chromatin domains (Figure 3 and Figure 4). In cases where the initial measurement is focused on individual genes, e.g., in RNA-seq data, the statistical assumptions are not violated. However, the potential association between adjacent genes, like those within a topologically associated domain (TAD), may exist. In such cases, the need for SAGO becomes less clear, as individual genes are measured independently, yet their underlying biology suggests potential associations through regulatory mechanisms. Both conventional statistics and SAGO can be considered valid approaches in these cases, and each may provide different insights. Conventional statistics identify enrichments of certain GO terms suggesting that genes from a specific category are enriched in the given condition. This enrichment might be due to genomic proximity or other regulatory mechanisms. By applying SAGO to such cases, we can investigate whether the identified enrichment is primarily driven by location-related mechanisms, which would be eliminated by SAGO, or by other regulatory processes, which would be retained. Thus, incorporating the TAD structure for intervals in RNA-seq data can be beneficial in distinguishing between spatial and other regulatory mechanisms.

In summary, the choice between conventional enrichment statistics and SAGO depends on the specific research question and the nature of the data. When dealing with large genomic domains, SAGO is necessary to account for spatial dependencies. However, when working with individual genes, both approaches are valid, and applying SAGO can help disentangle spatial effects from other regulatory mechanisms, providing valuable insights into the underlying biology.

Overall, SAGO offers a valuable approach to addressing spatial dependencies. SAGO thus enhances the accuracy of gene set enrichment analyses applied to various types of genomic data.

## Data Availability

The computational pipeline of SAGO and the script for creating the figures are available at: https://github.com/RacheliRap/SAGO_final/blob/main.

## Supplementary Materials

The following supporting information can be downloaded at: https://www.mdpi.com/article/10.3390/biology13030175/s1, Figure S1: SAGO corrects enrichments of terms with large residual values; Table S1: A full list of enriched GO terms in 400 randomly chosen genomic intervals; Table S2: SAGO results for the various datasets mentioned in the paper (each dataset in a different sheet).
