# Supplementary material for: A Cyclic Permutation Approach to Removing Spatial Dependency between Clustered Gene Ontology Terms
Source: Biology (Basel). 2024 Mar 8;13(3):175. doi: 10.3390/biology13030175 (PMC10967837; doi:10.3390/biology13030175)
Supplement: Supplementary file 1 [file biology-13-00175-s001.zip › Figure S1.pdf]

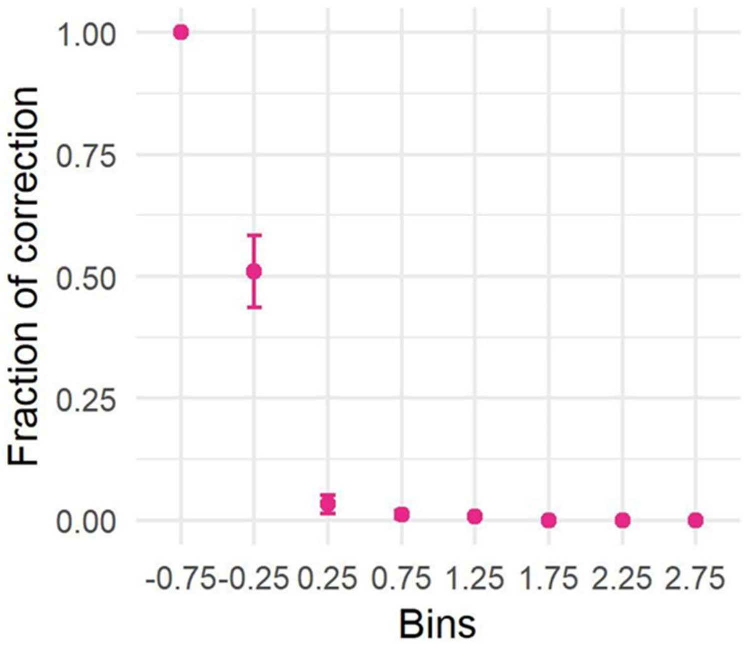

Figure S1. SAGO corrects enrichments of terms with large residual values. All enriched terms in the random runs shown in Figure 2e were organized in 8 bins based on their residual value ± 0.25 (X axis). The graph shows for each bin the fraction of terms (mean and standard error) that were corrected for spatial bias using the SAGO approach, as assessed across 100 random runs and employing Bonferroni correction for multiple hypothesis testing.
